# Ion beam figuring for X-ray mirrors: history, state-of-the-art and future prospects

**DOI:** 10.1107/S1600577524002935

**Published:** 2024-05-21

**Authors:** Riley Shurvinton, Hongchang Wang, Paresh Pradhan, Ioana-Theodora Nistea, Simon Alcock, Murilo Bazan Da Silva, Arindam Majhi, Kawal Sawhney

**Affiliations:** a Diamond Light Source Harwell Science and Innovation Campus DidcotOX11 0DE United Kingdom; IOM-CNR and Elettra-Sincrotrone, Italy

**Keywords:** X-ray optics, synchrotron radiation, ion beam figuring, X-ray mirrors

## Abstract

Synchrotron light sources require X-ray optics with extremely demanding accuracy for the surface profile, with less than 100 nrad slope errors and sub-nanometre height errors. Such errors are challenging to achieve for aspheres using traditional polishing methods. However, post-polishing error correction can be performed using techniques such as ion beam figuring (IBF) to improve optics to the desired quality. This work presents a brief overview of the history of IBF, introduces some of the challenges for obtaining such demanding figure errors, and highlights the work done at several in-house IBF facilities at synchrotron light sources worldwide to obtain state-of-the-art optical quality.

## Abstract 2

A review of the history of ion beam figuring and current developments is presented, with a focus on the production of high-quality X-ray optics.

## 1. Introduction

The optics used in modern X-ray synchrotron light sources have increasingly strict accuracy requirements. In order to maximize the focusing and intensity, reduce the spot size, and improve the energy resolution and coherence of the X-ray beam, height errors of less than 1 nm root mean squared (r.m.s.), slope errors of less than 100 nrad r.m.s., and a surface micro-roughness of 1 Å r.m.s. or better are required (Yamauchi *et al.*, 2011 ▸; Siewert *et al.*, 2012 ▸; Soufli *et al.*, 2012 ▸; Yumoto *et al.*, 2013 ▸; Chkhalo *et al.*, 2014 ▸; Idir *et al.*, 2014 ▸; Wang *et al.*, 2023*a* ▸). These errors must be maintained over active regions that may be several hundred millimetres in length, on mirrors that may have challenging elliptical or aspheric surface profiles.

Fulfilling these requirements is a challenge. Traditional shaping methods, such as grinding, lapping or diamond turning, are limited in their ability to deliver surfaces with the required quality, due to misalignment, vibrations or other inherent limitations (Rhorer & Evans, 2010 ▸). To overcome this, a final figuring step is typically employed after shaping and polishing to correct residual errors in the height and slope profile and converge to the strict requirements demanded for X-ray applications.

Ion beam figuring (IBF) is one such technique for post-polishing error correction. It is a non-contact method that uses a beam of energetic ions to gently sputter material from the target mirror, ensuring a stable and linear removal with minimal deterioration of surface micro-roughness. First proposed in the 1980s, IBF has matured and grown in prominence to meet the increasing demands of high-precision X-ray mirrors, aided by the increasing capabilities of metrology instruments to measure smaller errors with greater precision. Currently, IBF is used both commercially as part of the process chain of world-leading X-ray mirror suppliers and in smaller in-house plants to correct small numbers of X-ray mirrors to a high standard. Recent cutting-edge results demonstrate figuring of elliptical Si X-ray mirrors down to sub-0.5 nm r.m.s. height errors and sub-200 nrad r.m.s. slope errors (Wang *et al.*, 2023*b* ▸), with sub-100 nrad r.m.s. slope errors achievable for flat mirrors (Preda *et al.*, 2013 ▸). However, challenges remain for further reduction of these errors, involving improvements in the machine process to increase accuracy and reduce tolerance, combined with high-quality metrology, data processing and calculation.

This work presents a review of the IBF process for error correction of X-ray mirrors for synchrotron and free-electron laser (FEL) facilities. It begins with an overview of shaping, polishing and figuring techniques in Section 2, highlighting the importance of post-polishing figuring for X-ray mirrors. Section 3 gives a brief summary of the history and development of ion beam figuring, followed by an outline of the fundamental principles of IBF. Section 4 describes the challenges posed in obtaining sub-nanometre height errors and sub-100 nrad slope errors, and the solutions that have been proposed to tackle them. Finally, particular attention is called to the small ‘in-house’ IBF facilities that have recently been developed at various synchrotron light sources around the world. Section 5 concludes with a future outlook for the IBF technique, particularly in the field of synchrotron X-ray mirrors, and gives an update on the recent results of the in-house IBF plant at the Diamond Light Source, which has demonstrated sub-nanometre height error and sub-100 nrad slope error figuring for Si mirrors.

## 2. Background: optical figuring techniques

### 2.1. The limitations of mechanical shaping/polishing techniques for X-ray mirrors

**Figure 1:**
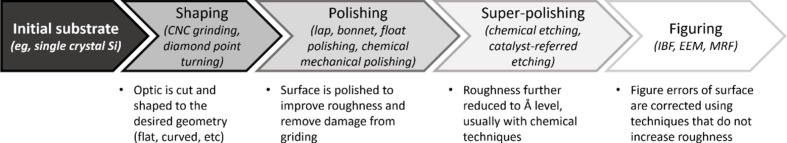
Manufacture process of X-ray mirrors. Figuring, *i.e.* post-polishing error correction, is performed as a final step after the mirror has been shaped and polished.

The process of manufacturing an optic from the initial material – such as producing an X-ray mirror from a single-crystal ingot of silicon – is typically done in multiple stages, as shown in Fig. 1 ▸. The initial step of shaping the mirror is generally performed using mechanical techniques, such as grinding, turning and lapping. A computer numerical controlled (CNC) system is usually employed for fast and accurate removal of material. For precision mirrors, single-point diamond turning (SPDT) is becoming increasingly important as a shaping and polishing technique, combining a high removal rate with a good precision (down to 100 nm figure error or better), even for complex geometries (Rhorer & Evans, 2010 ▸; Li *et al.*, 2011 ▸).

**Figure 2:**
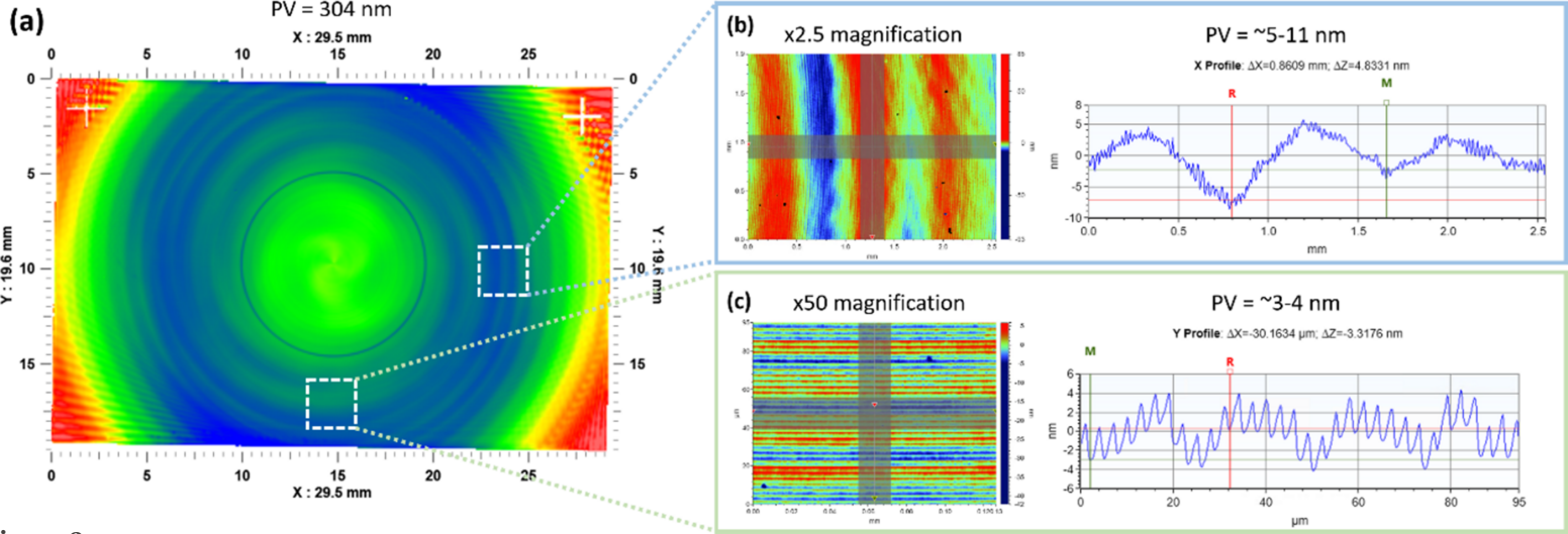
Impact of tool marks on a diamond-turned mirror. (*a*) The surface as measured using a stitching interferometer. (*b*, *c*) Magnified views of the surface measured using a micro-interferometer, revealing the impact of tool marks on both the millimetre and micrometre scale.

However, the figure accuracy of these mechanically shaped surfaces is limited, due to the stochastic nature of the process. Mechanically shaped surfaces will exhibit characteristic residual polishing or turning marks, which occur due to the finite size of the tooltip, tool wear, vibrations in the system related to the turning speed, errors in the motion or tolerance of the tool, as well as material effects or impurities (Li *et al.*, 2011 ▸; Beaucamp *et al.*, 2013 ▸). Fig. 2 ▸ shows an example of these marks on a mirror produced using SPDT, which occur at both the millimetre scale (damaging the figure error) and the micrometre scale (damaging the micro-roughness). Although these turning marks may only be a few nanometres in depth, they are sufficient to degrade the performance of the mirror for hard X-rays (Chon & Namba, 2010 ▸; Shinozaki & Namba, 2011 ▸).

After the initial shaping, the micro-roughness of mechanically shaped mirrors is generally insufficient for X-ray applications (Chon *et al.*, 2006 ▸). In the next step of manufacturing, the mirrors undergo several polishing stages, using mechanical or chemical removal techniques. The polishing first removes subsurface damage in the top layer of the mirror, and then successively improves the micro-roughness using mechanical or chemical techniques, with the latter step often referred to as ‘superpolishing’. From this, the micro-roughness of the mirrors may be reduced to the sub-Å levels required for X-ray applications. However, this polishing step only targets the micrometre-scale figure errors on the mirror’s surface. The millimetre-scale figure errors are not improved, and may even be increased by the polishing step depending on the geometry of the mirror (Li *et al.*, 2011 ▸). Thus, the polished mirror will have excellent roughness, but will exhibit residual figure errors in its height and slope that are unacceptable for high-precision applications such as on X-ray beamlines.

### 2.2. Deterministic figuring

Deterministic figuring techniques are typically the final step in optics manufacturing. This step is used to correct the millimetre-scale figure errors that remain on a polished mirror that has been processed using stochastic techniques, without negatively impacting the micro-roughness of the mirror. In deterministic figuring, the height or slope profile of the mirror is measured, and residual errors relative to the desired height or slope are identified and selectively corrected. The removal or deposition rates of deterministic figuring techniques is typically low, on the scale of nanometres per second, to allow for increased precision when correcting very fine errors. Most often, figuring involves removing material by either physical or chemical means; however, some techniques involve adding material to the surface in a controlled manner.

Three components are required for deterministic figuring. Firstly, high-quality information on the existing figure error of the mirror must be obtained, with a precision as good or better as the desired accuracy of the final figure. Secondly, the removal or deposition profile for the figuring method must be precisely known. Finally, there must be some way to selectively control the amount of material removed or added at different places on the mirror; for instance, by changing the speed of the tool procession, or by changing parameters of the figuring process to deterministically increase or decrease the removal/deposition rate.

**Figure 3:**
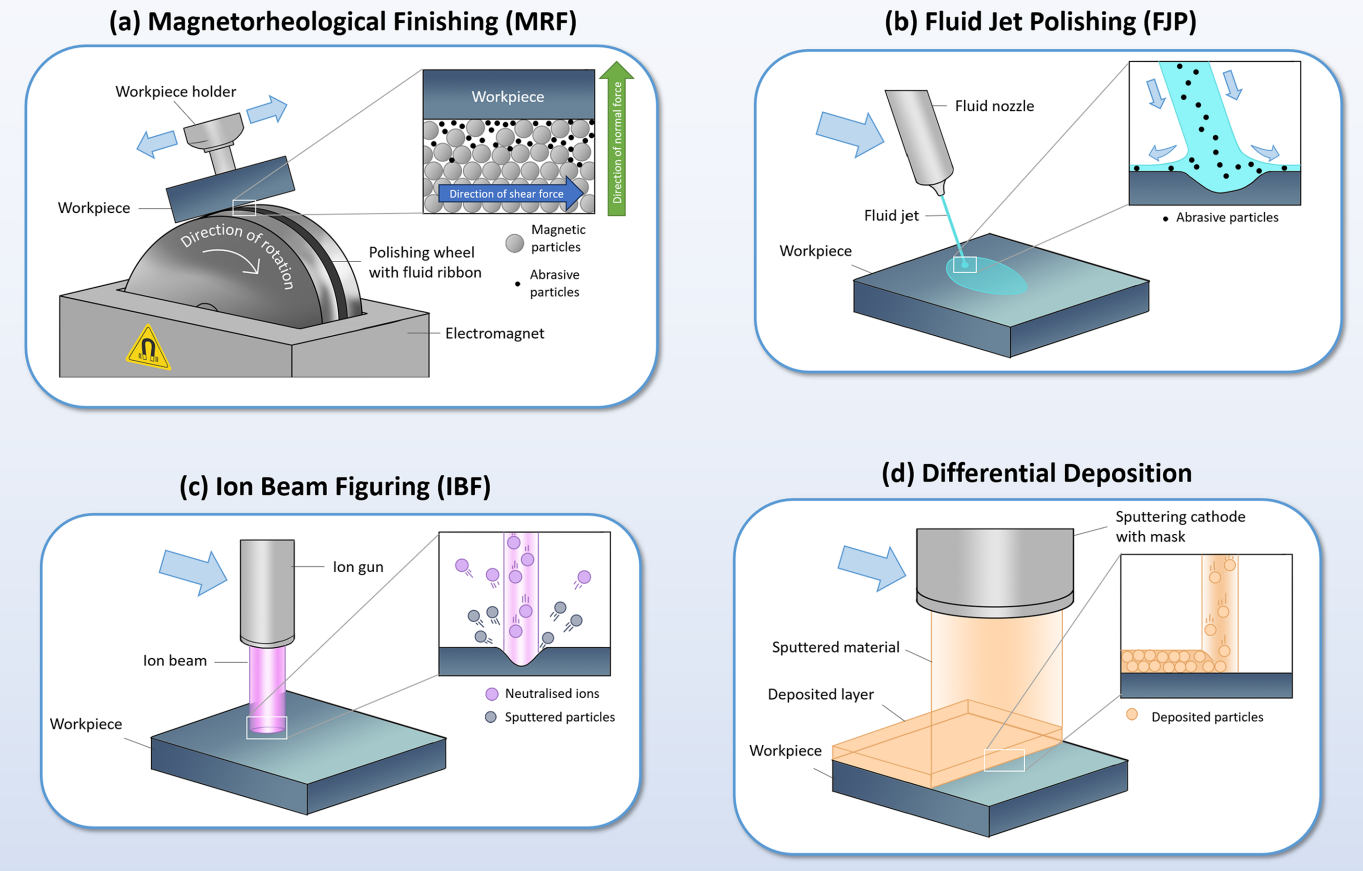
A schematic of four figuring techniques, which are used to correct height and slope errors on X-ray mirrors after polishing.

Currently, various techniques are used for deterministic figuring of X-ray mirrors. The techniques vary depending on whether material is added or removed, the removal or deposition method, and whether a polishing slurry is used. Prominent techniques include IBF, which uses a beam of neutralized ions to selectively sputter material from the mirror; magnetorheological finishing, a technique using a magnetic slurry which allows the fluid pressure to be controlled by an external magnetic field; fluid jet polishing/elastic emission machining, which uses a narrow high-pressure stream of polishing slurry to deterministically remove material and provide additional smoothing in a wider area; and differential deposition, which involves adding material using a sputtering source moved at variable velocity relative to the sample. A schematic of each of these techniques is shown in Fig. 3 ▸, and more details are discussed below.

#### 2.2.1. Magnetorheological finishing

Magnetorheological finishing (MRF) [Fig. 3 ▸(*a*)] is a polishing technique involving holding the mirror close to a rotating element (such as a wheel or belt) inside a magnetic field (Kordonski & Jacobs, 1996 ▸; Harris, 2011 ▸). A slurry flows between the mirror and the rotating element, containing a mix of magnetic particles and abrasive particles, and the action of the slurry against the mirror causes removal of material. The fluid pressure may be changed in real time by adjusting the magnetic field strength, which increases or decreases the viscosity of the slurry due to the presence of the magnetic particles. Thus, the removal rate may be adjusted at different points on the mirror’s surface, enabling deterministic correction. For precise deterministic polishing, the removal must be precisely known. Therefore, the impact of the fluid parameters – composition, consistency, temperature, pressure, and so on – must be carefully calibrated to ensure well controlled removal and precise error correction.

#### 2.2.2. Fluid jet polishing/elastic emission machining

Fluid jet polishing (FJP) and elastic emission machining (EEM) [Fig. 3 ▸(*b*)] are two closely related techniques, which use a high-pressure jet of fluid or polishing slurry that is rastered across the mirror to deterministically remove material (Yamauchi *et al.*, 2002*a* ▸,*b* ▸; Li *et al.*, 2011 ▸; Buss *et al.*, 2022 ▸). In addition to deterministic removal at the centre of the jet, a layer of fluid spreads in a wider area over the surface, which may contribute additional smoothing of the microscale surface features. Therefore, the technique maintains or may improve the microroughness of the surface along with correcting the figure error. Although the terms FJP and EEM are occasionally used interchangeably, generally EEM involves a chemical method of removal (Yamauchi *et al.*, 2002*a* ▸; Mori *et al.*, 2003 ▸), whereas FJP typically involves physical removal by the action of abrasive particles (Faehnle & Brug, 1999 ▸; Booij *et al.*, 2001 ▸).

FJP and EEM both allow high-precision correction of figure errors. The tool head size is typically on the order of 0.2–1 mm, allowing figure errors on a similar spatial wavelength to this to be corrected. The technique may be applied to a range of surfaces, and the parameters of the fluid jet, including pressure, nozzle size and the composition of the slurry, may be chosen depending on the requirements of the mirror. For this reason it is a very powerful and versatile technique. However, as with MRF, the impact of factors such as temperature, pressure and slurry composition (including species and particle size) must be exactly calibrated and controlled, both to ensure precise removal, and to minimize the impact on surface roughness of the jet (Fang *et al.*, 2006 ▸; Peng *et al.*, 2013 ▸; Li *et al.*, 2010 ▸). Additionally, due to the low removal rates, which vary depending on the hardness of the material from ∼10 nm s^−1^ to well under 1 nm s^−1^ (Faehnle & Brug, 1999 ▸; Yamauchi *et al.*, 2002*a* ▸; Li *et al.*, 2011 ▸), the total figuring time for EEM/FJP may be long, up to tens of hours or more. Hence, there is a particular challenge involved in ensuring the parameters are sufficiently stable to maintain accurate removal during the figuring.

#### 2.2.3. Differential deposition

Differential deposition [Fig. 3 ▸(*d*)] is an additive technique, which corrects errors by selectively depositing a thin film of material onto the mirror. The thickness of the layer is controlled by varying the motion of the sputtering source relative to the mirror (Alcock & Cockerton, 2010 ▸; Morawe *et al.*, 2019 ▸; Kim *et al.*, 2021 ▸, 2023 ▸; Bras *et al.*, 2023 ▸). A mask may be used to control the profile deposited on the mirror’s surface, which can vary in size from a few millimetres to tens of millimetres, enabling correction of both long- and medium-wavelength features. This technique is well suited to X-ray mirrors, and is applied as one or more corrective layers before deposition of the reflection coating typically used for beamline mirrors. As an additive technique, differential deposition is particularly well suited for correction of recessed or ‘negative’ features on a mirror’s surface, which are more challenging to correct with techniques that remove material. Careful consideration of the deposited material or material(s) is required to ensure the roughness of the substrate is not increased by deposition of the corrective layer (Morawe *et al.*, 2019 ▸; Kim *et al.*, 2023 ▸). In addition, the technique is generally restricted to 1D correction, which is effective for improving tangential height and slope errors for glancing-angle X-ray mirrors (such as focusing Kirkpatrick–Baez mirrors) but may be limited for 2D figuring.

#### 2.2.4. Ion beam figuring

Ion beam figuring [Fig. 3 ▸(*c*)] is a deterministic figuring technique which uses a neutralized ion beam to sputter material from the mirror. The size of the beam profile generally varies from below one millimetre to a few tens of millimetres, enabling both coarse and fine error correction. The beam typically has a very low removal rate, on the order of a few nanometres per second, allowing sub-nanometre figure errors to be obtained after correction. Some polycrystalline materials suffer surface roughness degradation after sputtering (Egert *et al.*, 1992 ▸); however, it is suitable for many materials commonly used for X-ray mirrors, such as Si, SiC, Zerodur and ULE glasses (Soufli *et al.*, 2012 ▸).

A crucial advantage of IBF is that there are no edge effects or fall-off seen at the edges of a mirror (Yang *et al.*, 2017 ▸), unlike for other methods such as MRF, as well as the majority of mechanical polishing techniques. Therefore, IBF is particularly suited to correct mirrors where the clear aperture is very close to the physical edges of the surface, and also strongly curved mirrors. Unlike MRF and FJP, a polishing slurry is not used, removing the requirement of post-processing cleaning and drying of the mirror.

Ion beam sputtering may also be used to improve surface micro-roughness when the beam is employed at an oblique angle (typically around 40°), in a process known as (direct) ion beam smoothing (IBS) (Frost *et al.*, 2004 ▸, 2009 ▸; Ziegler *et al.*, 2010 ▸; Arnold *et al.*, 2010 ▸). A related technique, known as ion beam planarization (IBP), involves an additional spin-coated layer which is chosen to have the same removal rate as the mirror underneath at a particular sputtering angle (Frost *et al.*, 2004 ▸; Arnold *et al.*, 2010 ▸; Li *et al.*, 2017 ▸). Both IBS and IBP have been shown to be promising for reducing the micro-roughness of surfaces, down to around 2 Å r.m.s. under optimal conditions. However, for Si X-ray mirrors, existing mechanical-chemical polishing techniques can already provide a surface with extremely low roughness (sub-Å r.m.s.). Hence, in this work, we will focus on IBF at normal or near-normal incidence for figure error correction.

## 3. History and principles of IBF

### 3.1. Development of IBF: from the 1980s to present day

The use of ion beams for figure correction of optics was first demonstrated in the late 1980s. Early works, such as those by S. R. Wilson *et al.* (Wilson & McNeil, 1987 ▸; Wilson *et al.*, 1989 ▸) and L. N. Allen *et al.* (Allen & Keim, 1989 ▸; Allen, 1989 ▸; Allen & Romig, 1990 ▸; Allen *et al.*, 1992 ▸) showed that it could correct figure errors down to several tens of nanometres r.m.s./peak-to-valley (PV), whilst maintaining a low surface micro-roughness for materials such as fused silica or ULE glass. Egert (Egert, 1992 ▸) further demonstrated that the technique was usable on a range of other materials without damaging surface micro-roughness, including silicon, germanium, sapphire and polycrystalline silicon carbide. However, significant degradation of micro-roughness was seen for certain polycrystalline metals, including aluminium and copper thin films.

Despite its potential uses, adoption of the technique was slow. This was due in part to limitations on the computing power of the day (Carnal *et al.*, 1992 ▸), and the comparative maturity of existing techniques for figure error correction. However, the advantages of this new technique were noted, such as its application to large optics (metre-scale), gentle removal rate, and the lack of edge falloff effects seen for some other correction techniques.

IBF began to see more interest during the early 2000s, as computing and metrology technology evolved and the demands of third-generation synchrotron light sources such as BESSY-II and SPring-8 became apparent (Ishikawa *et al.*, 2001 ▸; Yamauchi *et al.*, 2002*a* ▸; Schindler *et al.*, 2003 ▸). Work to advance and commercialize the IBF process (alongside other related processes, such as reactive ion beam etching (RIBE) and IBP] was led by a research collaboration including the Liebniz Institute of Surface Engineering (IOM), Liepzig University, Nanotechnologie Liepzig (NTGL) and Neue Technologien (NTG) (Schindler *et al.*, 2003 ▸; Jacobs, 2004 ▸; Hänsel *et al.*, 2007 ▸; Franz & Hänsel, 2010 ▸). Commercial IBF plants were developed with the capability to figure optics of up to 700 mm and reduce r.m.s. errors to the nanometre or even sub-nanometre range. At the time, the development was largely driven by demands for high-quality mirrors in use of semiconductor lithography, operating in the high-energy extreme ultraviolet (EUV) (Franz & Hänsel, 2010 ▸). However, there was also discussion on the use of the technology for developing X-ray mirrors.

In the 2010s and early 2020s, the prominence and capabilities of IBF continued to evolve. In a 2018 review, D. Schaefer described IBF as ‘a well known finishing technique for the production of ultra-precise optical surfaces’ (Schaefer, 2018 ▸). Following the development and maturation of IBF for figuring EUV mirrors, there was an interest from the X-ray community in adopting the technique for high-quality finishing of X-ray mirrors – particularly curved mirrors, due to its superior handling of edges. Notable companies such as ZEISS (Thiess *et al.*, 2010 ▸) and Thales SESO (Peverini *et al.*, 2020 ▸) also began to employ IBF as a finishing step on their mirrors, enabling production of extremely high-quality X-ray mirrors. In particular, Thales SESO reported in 2020 the use of IBF to produce a 200 mm mirror with a 1D slope error of below 100 nrad, which is a crucial milestone of optical quality in the X-ray regime.

Over the past decade, many laboratories and synchrotron light source facilities have also begun to advance ‘in-house’ IBF plants. A key advantage of these in-house plants is that they can take advantage of the cutting-edge metrology facilities housed in many synchrotron or XFEL laboratories, which are often as good, if not better, than those of commercial optical manufacturers, allowing rapid and efficient convergence of figure errors on X-ray mirrors after several IBF iterations. Works by C. Liu *et al.* at the Advanced Photon Source (Liu *et al.*, 2015 ▸), M. Hand *et al.* at the Diamond Light Source (Hand *et al.*, 2019 ▸) and M. Idir *et al.* and T. Wang *et al.* at the National Synchrotron Light Source (Idir *et al.*, 2015 ▸; Wang *et al.*, 2019 ▸) all present IBF projects for 1D or 2D figuring of X-ray mirrors. L. Zhou *et al.* (Zhou *et al.*, 2007 ▸) present an IBF system at the National University of Defence Technology; and Y. Zhang *et al.* (Zhang *et al.*, 2022 ▸) present an IBF system at Tongji University which was used to produce mirrors for the Shanghai Synchrotron Radiation Facility. These facilities have reported notable results in recent years, including a recent demonstration from the National Synchrotron Light Source of sub-0.5 nm r.m.s. height error and sub-200 nrad r.m.s. slope error over a 150 mm-long clear aperture (Wang *et al.*, 2023*b* ▸).

**Figure 4:**
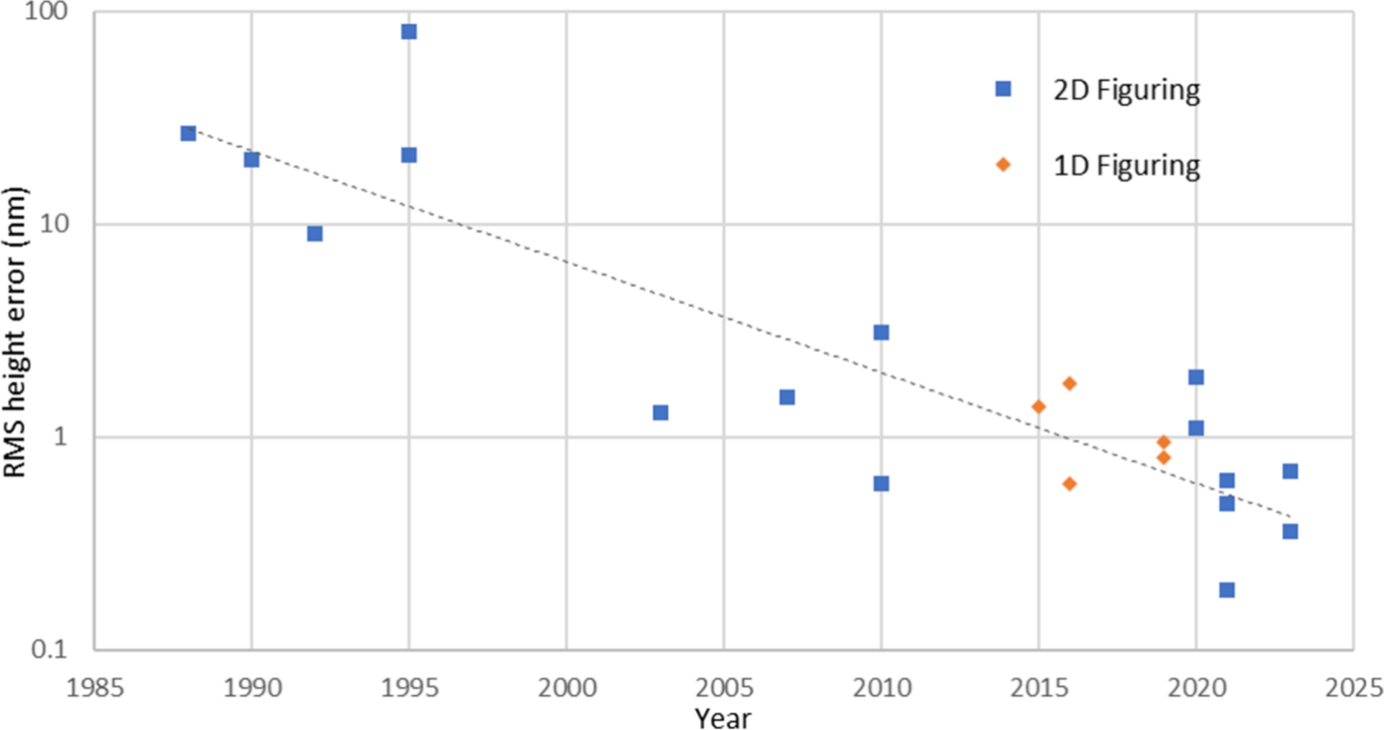
A summary of results from the literature of successively lower height errors obtained using IBF, from the late 1980s to the present day. Since the inception of the technique, advances in metrology techniques and the maturation of IBF technology have greatly improved the process, allowing the achieved r.m.s. errors to half approximately every five years. In recent years, the technology can reliably provide sub-nm height errors for 2D surfaces. See Appendix *A*[App appa] for a chronological list of references.

Fig. 4 ▸ shows a graphical summary of the development of IBF, and the achievement of successively lower height errors over time.

### 3.2. Principles of ion beam figuring

In brief, an IBF system requires two components: an ion source, and a motion stage capable of performing a raster scan. When the ion beam hits the surface of the mirror, material is sputtered at a rate that is linearly proportional to the dwell time. By scanning the beam at variable speed across the surface, material may be deterministically removed in a way that is highly precise and controllable. IBF is typically carried out within a vacuum, in order to maximize the mean free path of the ions and minimize the presence of contaminants and reactants. Therefore, the components of the IBF system are housed within a vacuum chamber, equipped with pumps to allow it to reach the desired vacuum pressure of ≤1 × 10^−4^ mbar (with a typical working pressure of ∼1 × 10^−4^ mbar). The individual components of an IBF system are detailed below.

#### 3.2.1. Ion source

The parameters of the ion source dictate the removal capabilities of the IBF system. Temporal stability and linearity of the removal are crucial for precise figuring, and therefore the ion source must also be highly stable.

A Kaufmann (gridded) ion source is typically used, with the grids generally made of pyrolitic graphite due to improved thermal stability. Argon is the most common choice of gas, although other heavier inert gases such as neon (Chkhalo *et al.*, 2017 ▸) or xenon (Chkhalo *et al.*, 2014 ▸) are also used, which may improve the resulting surface roughness for some materials. When inert gases are used, the sputtering process is purely physical, which helps to ensure linearity of the removal rate.

In RIBE, a variant of IBF, reactive gases such as oxygen are used for sputtering, causing the formation of chemical products during the etching process (Bauer *et al.*, 2017 ▸). RIBE has the advantage of higher removal rate in certain cases (Idir *et al.*, 2013 ▸), and can be more applicable to certain materials, such as aluminium alloys. However, the chemical reactions caused at the surface involve a much more complicated removal process compared with IBF using inert gases. Hence, a significant challenge for RIBE is the precise calibration and stability of the removal rate.

Bombardment of a mirror surface by high-energy ions can cause surface damage and pitting, which increases the micro-roughness and degrades the quality of the mirror (Makeev *et al.*, 2002 ▸). Therefore, the beam energy must be carefully chosen to ensure gentle removal and minimize surface damage. The optimal energy depends on the species and mass of the ions used, as well as the material of the mirror being figured. For Ar ions, the typical beam energy for IBF is between 400 and 1000 eV, with a resulting removal rate of ∼1 nm s^−1^ for Si mirrors.

The ion beam may be focused or collimated depending on the beam optics, with both having individual advantages and disadvantages. Focused ion beams allow for a higher current density, and therefore a higher removal rate. In comparison, a collimated ion beam typically produces a larger beam profile, which may be more appropriate for coarse correction, but with a lower peak current density. If an additional aperture is used to filter the beam, the resulting shape will vary from a Gaussian profile at large distances to a top-hat shape close to the aperture. The angle of incidence of the ion beam on the mirror must also be considered, particularly for strongly curved mirrors (Liao *et al.*, 2014*a* ▸,*b* ▸). This can be controlled using a five- or six-axis motion system to ensure the beam incidence remains normal to the mirror throughout figuring. Alternatively, it can be post-compensated by considering a dynamic removal function at different points on the surface (Gu *et al.*, 2023 ▸) (see Section 4.3 for further discussion).

#### 3.2.2. Motion configuration

**Figure 5:**
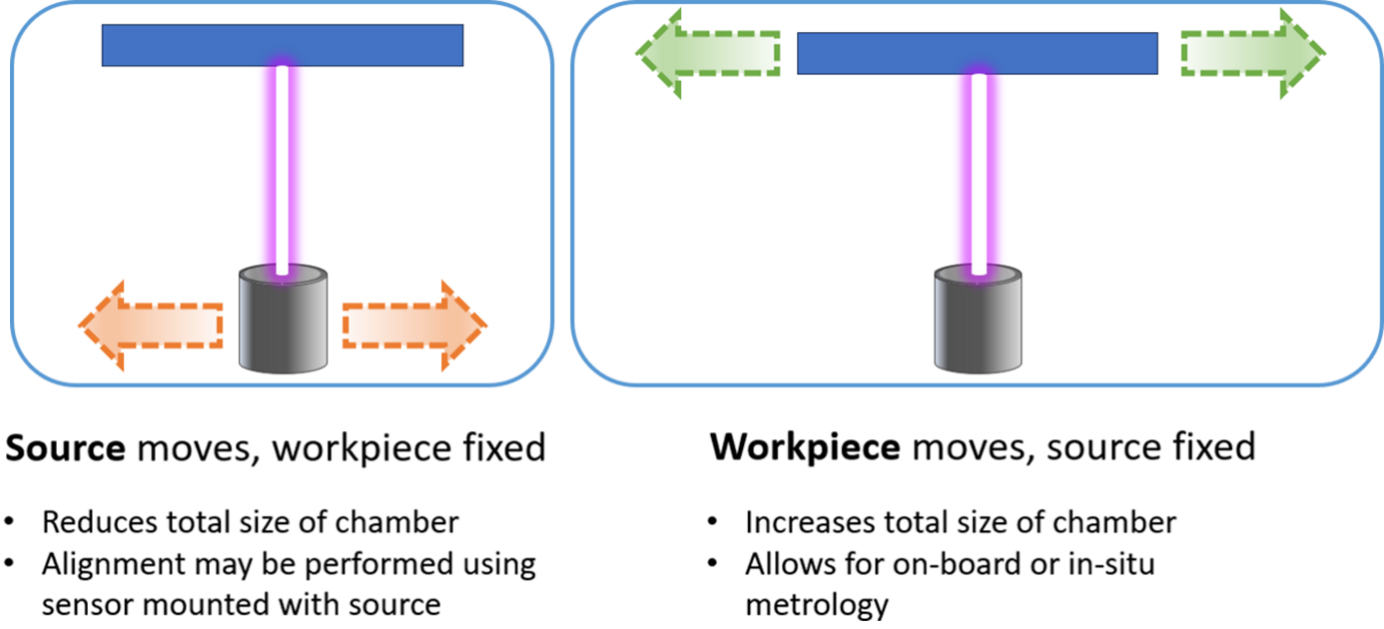
Sketch of the two possible motion setups, where either the source (left) or the mirror (right) is mounted on a motion stage, and the other component is fixed in place during figuring.

During IBF correction, one of either the mirror or the ion source may move, while the other component is fixed (Fig. 5 ▸). A third approach is to fix both the source and the mirror, and use a movable mask/aperture to control the removal (Peverini *et al.*, 2010 ▸; Preda *et al.*, 2013 ▸), but this technique has not been widely adopted.

Moving the ion source has the advantage of reducing the overall size of the IBF plant. In configurations where the mirror position is fixed, the vacuum chamber only needs to be as long as the size of the largest mirror to be figured; whereas, if the mirror moves, the vacuum chamber must be twice as long as the size of the largest mirror to accommodate its length as it moves from one side of the source to the other. The volume of the chamber has important implications for how long it takes to be pumped to vacuum, as well as practical considerations around the overall size of the IBF plant. On the other hand, fixing the ion source position and moving the sample allows for much more flexible use of on-board metrology during the process, as discussed in Section 3.2.3.

The required motion of the ion beam to correct a given surface is initially calculated as a dwell time map, consisting of short increments of time at discrete positions on the surface. However, trying to implement this in a literal way would require discontinuous motion with infinite velocity between the dwell positions. Instead, the position–time scheme is typically adapted to a position–velocity–time (PVT) scheme, allowing the velocity of the motor to be smoothly modulated (Wang *et al.*, 2020*a* ▸). Additional care may be needed to ensure the acceleration is also smooth, and that the maximum acceleration and velocity during motion do not exceed the limits of the motion stage (Wang *et al.*, 2023*c* ▸).

The number of axes of motion required depends on whether 1D or 2D figuring is being performed. 1D figuring involves only one axis of motion relative to the ion beam, and allows the correction of mirror surfaces over a narrow aperture of a few millimetres wide. This is suitable for figuring of grazing-incidence mirrors, such as Kirkpatrick–Baez (KB) focusing mirrors (Zhou *et al.*, 2016*a* ▸) using a very simple setup. Conversely, 2D figuring involves scanning the beam in two dimensions, enabling greatly improved flexibility but at the cost of increased complexity. 2D figuring plants require at least two axes of motion, and typically use between three and six axes (including manipulation of source-sample distance, and rotation of the sample with respect to the beam), as well as requiring significantly more complex calculations to implement (see Section 4.3 for more discussion).

#### 3.2.3. On-board metrology

**Figure 6:**
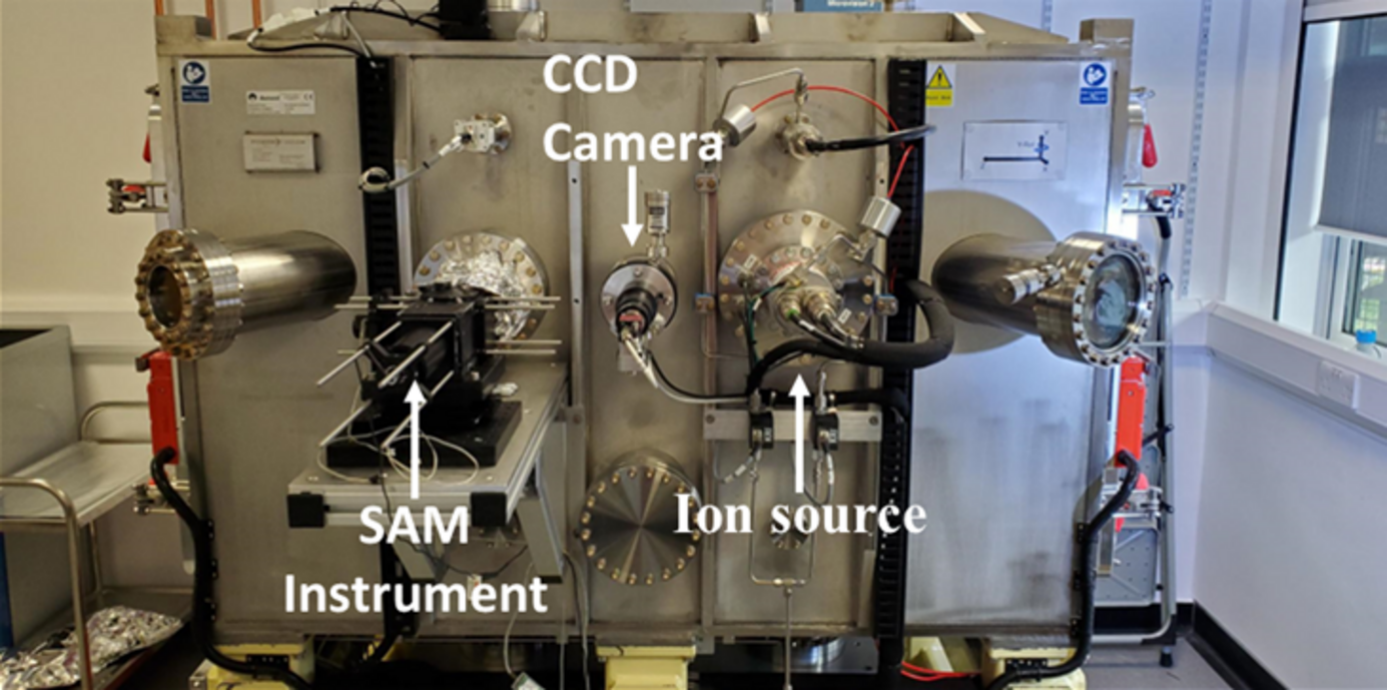
A photograph of the IBF plant at the Diamond Light Source, which is equipped with a fixed ion source, a CCD camera for mirror alignment and targeting, and a SAM instrument for measuring the curvature of the mirror. Figure reproduced with permission from Mahji *et al.* (2024 ▸).

One novel advantage of IBF is its compatibility with on-board metrology, as demonstrated by the in-house IBF facility at the Diamond Light Source (Hand *et al.*, 2019 ▸) (Fig. 6 ▸). Various optical measurement devices may be installed in an IBF plant, aiding in either sample targeting and alignment, or allowing measurement of the height or slope profile of the mirror under figuring. A straightforward example of a targeting aid is a camera, which may be used to find the position of the edges or fiducial marks on a mirror. Other options to measure the position and orientation of the mirror may include an on-board autocollimator for angular calibration. In the Diamond Light Source IBF, an autocollimator was used for calibration of the angular motion stage, and to characterize rotation errors caused by the translation stage during commissioning (Hand *et al.*, 2019 ▸).

For measurement of the height or slope profile of a mirror within the IBF chamber, a wide range of optical metrology instruments are suitable for on-board installation. For instance, the Diamond Light Source in-house IBF facility is equipped with a speckle angular measurement device (SAM), enabling measurement of the curvature of a mirror using stitching speckle deflectometry (Sutter *et al.*, 2011 ▸; Wang *et al.*, 2021*a* ▸; Mahji *et al.*, 2024 ▸). On-board measurements have the advantage of being carried out in vacuum, helping to reduce air fluctuations from pressure and temperature. They also allow rapid measurement of a mirror, compared with *ex situ* measurements (which require venting the IBF vacuum chamber to atmosphere, removing the sample to perform the measurements, and then subsequently pumping the chamber down to vacuum again, which is a highly time-consuming process).

Fundamentally, the precision of on-board metrology instruments is limited, due to environmental effects such as vibrations, and also clamping (see Section 4.2) and heating/cooling (see Section 4.1.4). To converge to the required sub-100 nrad accuracy, *ex situ* metrology must be used to give the required measurement precision. However, on-board metrology can be used for initial coarse corrections of a mirror, when the slope errors may be on the scale of hundreds of nanoradians or even several microradians. Additionally, it may be used to extract beam removal function (BRF) parameters and removal rate of the ion beam from an etched crater (see Section 4.1.2). Both of these applications can speed up the IBF process significantly, compared with relying exclusively on *ex situ* metrology.

## 4. Challenges for accurate error correction

In principle, the IBF process is reasonably simple and straightforward. However, to obtain mirrors with the extremely low height and slope errors required for current-generation X-ray light sources, there are several challenges that must be overcome. Obtaining and processing high-quality metrology data of the surface under correction with the necessary precision requires a careful and thoughtful approach. Using these data to calculate the optimal dwell time to correct a given surface profile is also not a trivial task, and different approaches should be considered for different surfaces. Finally, in order to ensure accuracy of the figuring, all elements of the IBF process, including the coordinate targeting and fiducialization, and characterization of the ion beam removal, must have their tolerances and uncertainties carefully considered and reduced as much as possible.

### 4.1. Technical challenges of the IBF process

#### 4.1.1. Coordinate alignment and targeting

The IBF process relies on alignment between (at least) two coordinate systems: the IBF coordinate system, describing the position of the ion beam relative to the mirror; and the metrology coordinate system, containing the measured height or slope information about the mirror. Accurately correcting errors on a millimetre-scale spatial wavelength requires position and alignment tolerances of 100 µm or less, and any larger misalignment dramatically reduces the accuracy of the figuring process (Wang *et al.*, 2023*b* ▸).

A common approach to coordinate alignment is to use a ‘fiducial mark’, typically a small cross, made on the surface of a mirror. Coordinate systems may then be aligned relative to the centre of this feature. For example, in an IBF setup equipped with an onboard camera, the camera can be used to find the sample position by locating the fiducial mark. An instrument that measures the height profile of the mirror, such as a stitching interferometer, will also be able to resolve the mark, allowing alignment between the two coordinate systems. For metrology instruments that measure slope or curvature, a suitable fiducial mark can instead be obtained by etching a crater or a furrow on the mirror using an ion beam. The measured location of these etched marks can be directly compared with the ion beam targeting coordinates, provided the mirror is returned to the exact same position in the IBF chamber after *ex situ* measurement (Zhou *et al.*, 2016*b* ▸; Hand *et al.*, 2019 ▸). A combination of cross and crater fiducial marks may be used to allow comparison between various metrology instruments.

#### 4.1.2. Accurate determination of beam removal function

To ensure a high accuracy of material removal during IBF, the removal rate and the shape and size of the ion beam should be determined to a high accuracy. This is usually done by etching a crater on a mirror, and then fitting the resulting measured height profile to a two-dimensional analytical function *B*(*x*, *y*), the BRF. As the removal process is generally highly linear, the material removal per unit time can be obtained by dividing the amplitude of the measured crater by the number of seconds it was etched for.

**Figure 7:**
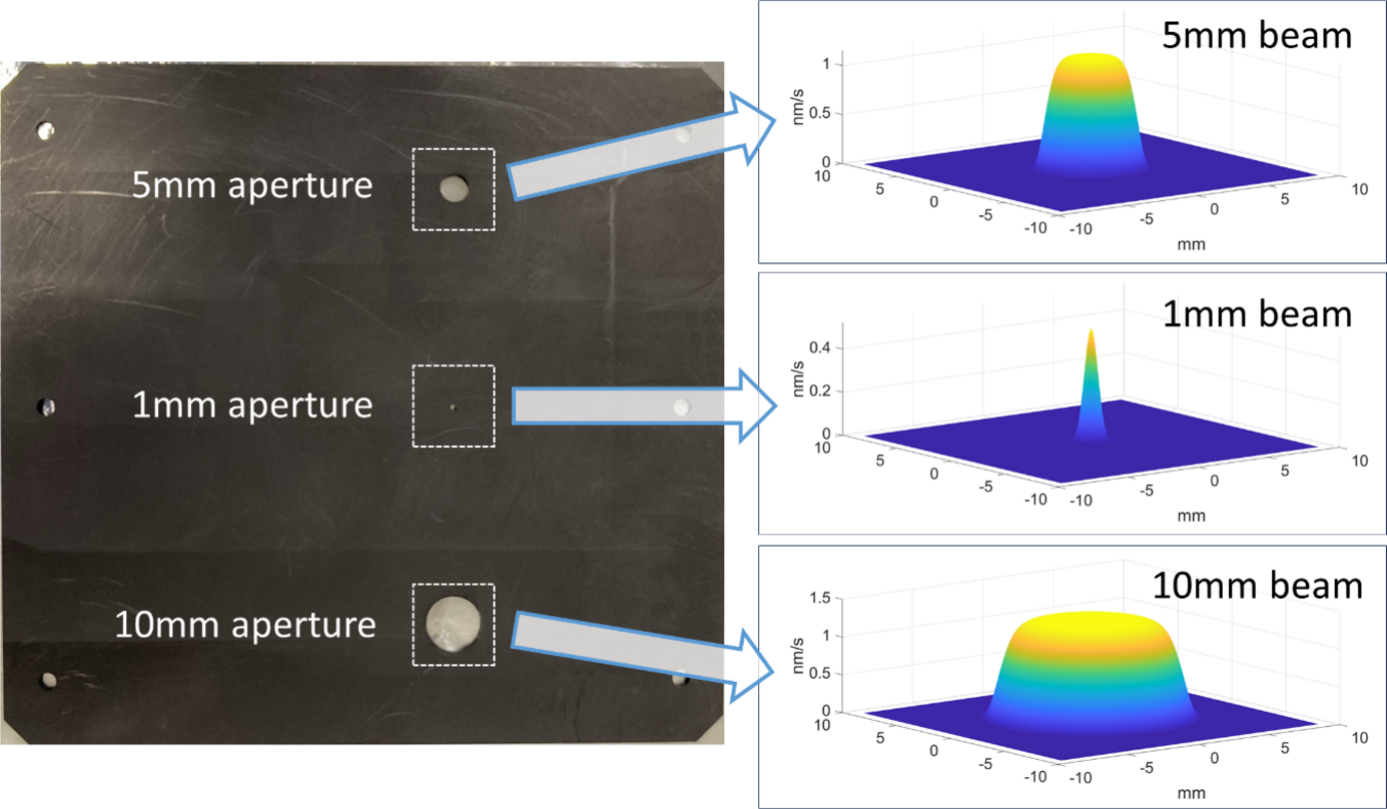
Left: an aperture plate used in the IBF plant at Diamond Light Source, with 1 mm, 5 mm and 10 mm diameter circular apertures that may be used to selectively mask the ion source during figuring. Right: the removal profiles obtained from each aperture, derived from fitting to measured data with either a Gaussian or super-Gaussian function.

Fig. 7 ▸ shows a graphite aperture plate used to select a smaller region of the ion beam for etching, and the resulting BRF obtained for each aperture. For smaller apertures or when a focused source is used, the BRF will typically be Gaussian in shape; for larger apertures in front of a collimated source, the BRF will instead tend to take a ‘top-hat’ shape described by a higher-order super-Gaussian function. The BRF will be influenced by the distance between the mirror surface and the ion source/aperture plate, and also by the angle of incidence [which is particularly relevant for curved mirrors (Liao *et al.*, 2014*a* ▸)]; the material and structural composition of the mirror; and the history of the mirror, due to oxide formation and depth, total removal depth, and other effects. Where possible, a BRF obtained from a crater etched directly on the mirror under figuring will give the most accurate determination of removal for that mirror surface. However, BRFs may be obtained by etching on ‘sacrificial’ test mirrors with reasonable accuracy, especially for the initial stages of coarse figuring.

#### 4.1.3. Choosing optimal tool shape and size

**Figure 8:**
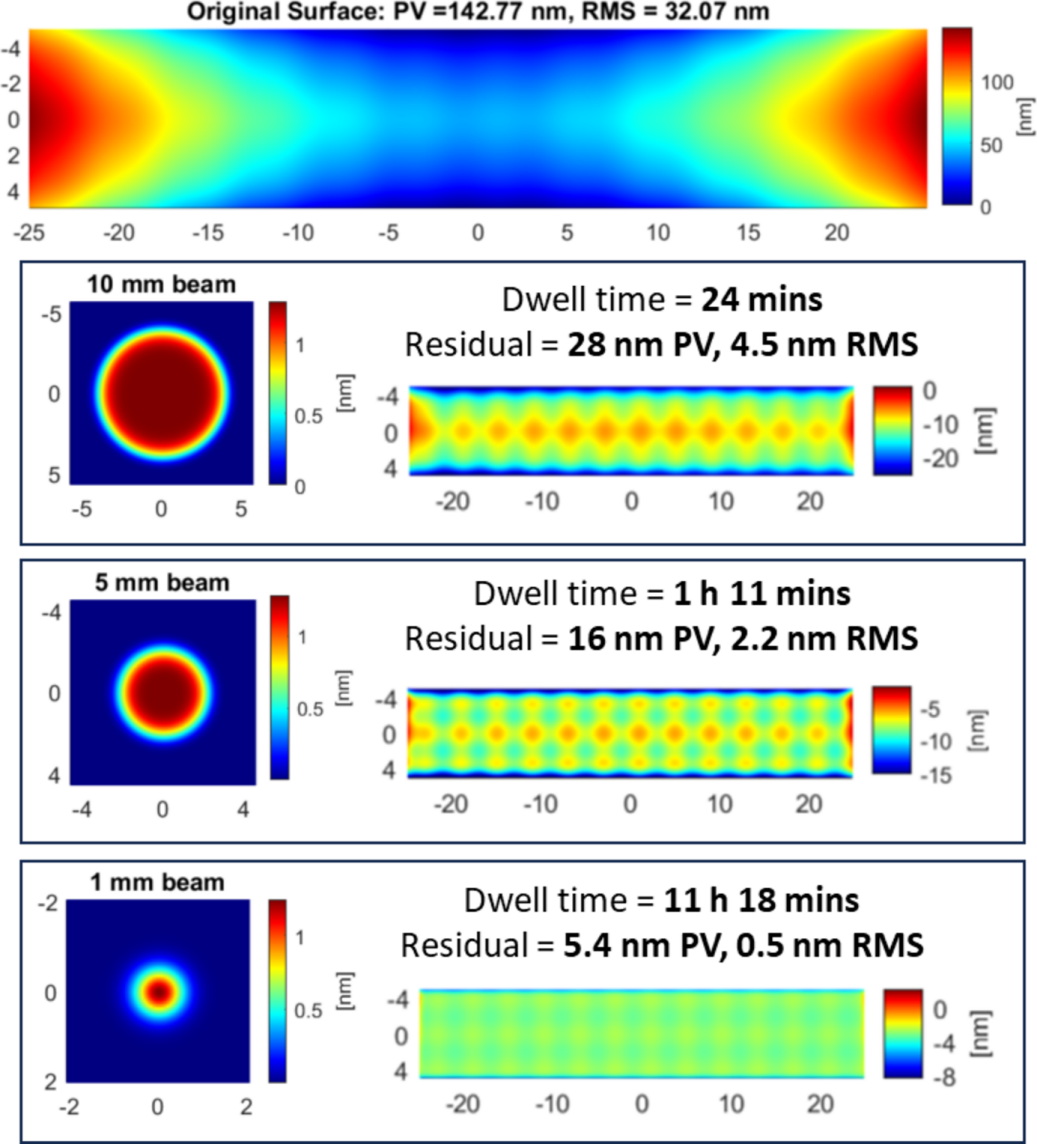
The impact of beam size for correction of an analytical input surface (top), comprising a low-frequency parabolic component with a period of ∼100 mm and a medium-frequency sinusoidal component with a period of ∼3 mm. The 10 mm and 5 mm beams are effective at correcting the low-frequency errors with a short total dwell time. However, they cannot correct the medium-frequency errors, which remain visible in the residual surface post-figuring. The 1 mm beam effectively corrects both the low-frequency and the medium-frequency errors, yielding a residual surface with greatly improved r.m.s. and PV errors. However, the required dwell time is much longer than for the larger beam sizes.

Use of an aperture plate (Fig. 7 ▸) to selectively mask out part of the ion beam allows the beam size and shape used during figuring to be carefully controlled. Typical sizes range from 10 mm or more in diameter to as small as 0.5 mm for focused sources. The appropriate tool size and shape chosen depends on the nature of the errors which are to be corrected. In general, beam size represents a trade-off between reducing total dwell time (associated with larger beam sizes) and minimizing residuals (associated with smaller beam sizes) (Allen & Keim, 1989 ▸). Fig. 8 ▸ shows an example of the impact of beam size for correcting a measured mirror surface.

In approximate terms, a Gaussian beam with a diameter *d* can effectively correct errors with a spatial period ≥2*d* (Zhou *et al.*, 2008 ▸; Arnold *et al.*, 2010 ▸). When the beam size is too large to effectively correct the errors present on the surface, the overall residuals will be higher, and the convergence of the figuring will worsen. However, when the beam size is small compared with the low-frequency errors present on a surface, the dwell time needed for figuring may be increased to tens or even hundreds of hours. Such long dwell times are not generally feasible and will cause errors due to source instability, sample heating and expansion, *etc*., leading to poor convergence (Wang *et al.*, 2022 ▸). As such, figuring is usually performed in multiple iterations, starting with coarse correction using a larger beam and progressing to finer corrections using smaller beams until the desired figure accuracy is achieved. This ensures the dwell time for each iteration is kept acceptably low, but at the cost of increasing total processing time, due to the need to measure the surface profile after each iteration.

#### 4.1.4. Mirror heating and thermal expansion

**Figure 9:**
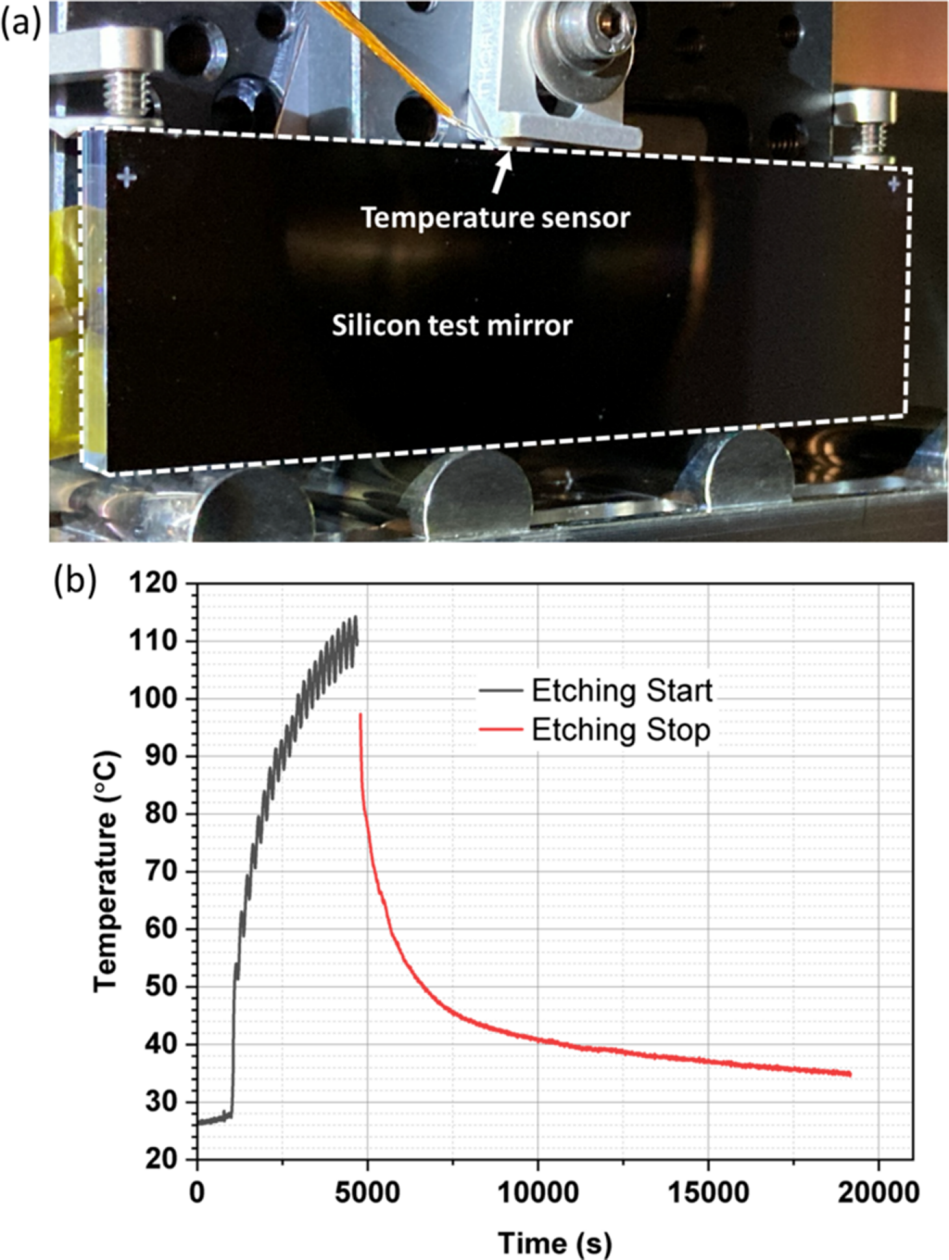
(*a*) Photograph of the Si mirror used for the temperature test. A temperature sensor was installed at the top of the mirror (labelled). (*b*) Graph of temperature over time when a raster path was etched on the sample with the ion beam, with a total etching time of 1 h and 5 min. The sample temperature increased dramatically during etching by over 80°C, followed by a rapid fall after etching completed.

One potential issue associated with long dwell times during figuring is heating of the mirror. The ion beam imparts heat to the mirror as it passes over the surface, and this heating is larger when a focused source is used, the source–sample distance is low, and there is poor thermal conductive contact between the mirror and the holder/chamber to allow cooling. Depending on conditions, the temperature of the mirror may increase by tens or even hundreds of degrees Celsius during figuring. A test performed at the IBF plant at Diamond Light Source showed that, during an hour-long correction using a 5 mm beam from a collimated ion source, the temperature of a Si mirror increased by over 80°C, before rapidly cooling after the figuring was completed and the ion source switched off (Fig. 9 ▸).

This heating effect may cause significant expansion and/or change in curvature of the mirror under figuring. For example, given an increase of 100°C, a Si mirror 300 mm in length will expand by 78 µm, which is non-negligible considering the tolerances required for IBF. Depending on the clamping of the mirror, this thermal expansion may cause the curvature of the mirror to change, sometimes dramatically, which can also impact the figuring process. Additionally, other components in the chamber, such as the sample holder, may be made of materials such as Al or stainless steel with significantly higher thermal expansion coefficients. Expansion of these components due to high temperatures can cause further strain, deformation or potential misalignment of the mirror. Finally, high temperatures can also change the linearity of the sputtering process, reducing the accuracy of figuring.

For IBF, the heating of the mirror during figuring should therefore be minimized. This may involve reducing the total dwell time wherever possible; performing figuring in multiple short cycles and allowing the mirror to cool in between cycles (Wang *et al.*, 2023*a* ▸,*b* ▸); or adjusting the design of the sample holder and mount to allow for improved passive cooling (*e.g.* using thermal braids).

### 4.2. Metrology

#### 4.2.1. High precision and repeatability requirements

Key to the ability of an IBF process to obtain sub-nanometre height errors and sub-100 nrad slope errors is a metrology system capable of accurately measuring such errors. For successful correction of figure errors, it is imperative to accurately measure the surface profile before and after figuring. The repeatability of metrology must be at least as good as the lowest errors sought, and preferably better. Much of the development of the IBF process has moved hand-in-hand with the development of metrology systems to reliably measure smaller features on optical surfaces with greater precision. As the tolerances and uncertainties of an IBF system must be minimized to produce mirrors with such low figure errors, so too must the metrology instruments be carefully calibrated and sources of random and systematic measurement errors be eliminated.

Each metrology instrument will typically measure either the height, slope or curvature of the test surface. Conversion between any such measurements can be achieved using numerical calculus. Algorithms should be chosen to ensure numerical errors are minimized compared with the measured data. However, care must be taken; integrating a slope measurement to obtain a height profile can significantly magnify any noise or errors, whereas differentiating a height measurement to obtain slope involves careful consideration of the window used to calculate slope and the filters (if any) applied to the data before calculation. In general, a thoughtful approach is needed when comparing measurements from different instruments.

X-ray mirrors typically involve an active area much longer than it is wide. As such, stitching methods are common when measuring the height or slope profile. This involves taking a large number of overlapping sub-aperture measurements of the surface, using a device such as an interferometer (Yamauchi *et al.*, 2003 ▸), that are then combined or ‘stitched’ to form a view of the entire surface. However, stitching sub-apertures is nontrivial, and solving the optimization problem to combine individual apertures takes a large amount of processing power.

Options for measuring the slope profile include instruments such as the long trace profiler (Rommeveaux *et al.*, 2008 ▸; Takacs *et al.*, 1987 ▸) and the nanometre optical component measuring machine (Siewert *et al.*, 2008 ▸), both of which were designed to measure the tangential slope of glancing-angle mirrors like those often used in X-ray beamlines (with sagittal slope information obtained in only a narrow area). Both approaches yield 1D slope profiles, but 2D slope information may be obtained using a raster scan over the mirror surface.

In addition to the *ex situ* metrology described above, *in situ* at-wavelength metrology can also be used to assess the height and slope errors of an X-ray mirror. Such at-wavelength metrology is extremely powerful for assessing the performance of a mirror in its working conditions with high precision (Berujon *et al.*, 2014 ▸; Wang *et al.*, 2014 ▸, 2015*b* ▸; Yamauchi *et al.*, 2011 ▸), with sensitivity of a few nanoradians achievable (Wang *et al.*, 2015*a* ▸,*b* ▸). An IBF plant installed on an X-ray beamline, such as that presented by Preda *et al.* (2013 ▸), could further integrate the process with *in situ* measurement and diagnostics. This would allow even more rapid convergence of the IBF process to highly accurate results.

As well as the millimetre-scale figure errors, it is also important to monitor the micrometre-scale features of a mirror. Excessive micro-roughness of mirror surfaces, generally >0.3 nm r.m.s. for X-ray mirrors, causes scattering and loss in intensity of the reflected X-ray beam. Therefore, it is essential to ensure that the micro-roughness of the mirrors is not increased during the figuring process. Techniques for characterizing micro-roughness include micro-interferometry; tactile stylus contact measurement; scanning probe techniques, such as atomic force microscopy (AFM); and X-ray reflectometry (XRR). Notably, techniques like micro-interferometry and AFM only measure a small region of the sample. Therefore, it is important to ensure that representative parts of the surface are chosen, and it is good practice to measure at several different positions.

#### 4.2.2. Clamping stresses and sample position alignment

**Figure 10:**
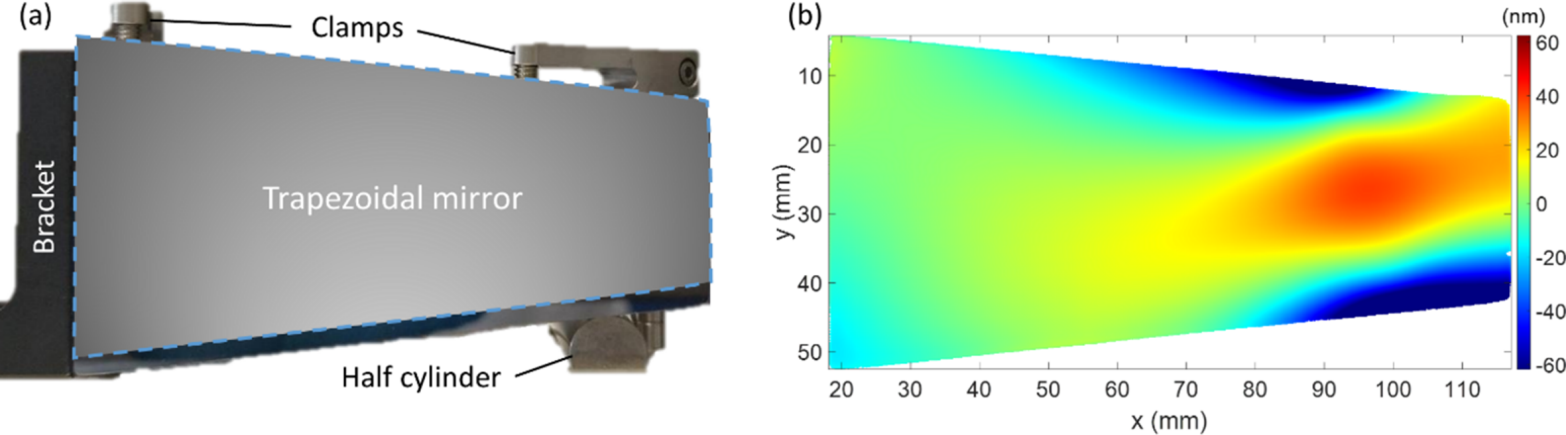
(*a*) A photograph of a trapezoidal mirror, clamped in preparation for figuring with IBF. (*b*) Height profile showing the deformation of the mirror in this clamping configuration, obtained as the difference between the measured height profile when clamped, and the measured height profile when free-standing. A large deformation is seen of more than 100 nm, which is most severe at the thinner end of the mirror, indicating the force applied by clamping significantly impacts the height profile of the mirror.

A key aspect of accurate metrology is minimizing the mechanical stress and gravitational ‘sag’ deformation when a mirror is measured. Depending on the thickness of the mirror, deformation caused by improper clamping could be tens or even hundreds of nanometres, as demonstrated in Fig. 10 ▸. Therefore, wherever possible, a mirror should be measured under as little force as possible, and ideally free-standing. This allows the stresses on the mirror to be kept as consistent as possible during each measurement.

This requirement to minimize force on the mirror during metrology poses a challenge for the IBF process in terms of the sample positioning. Correction of a mirror often progresses over many iterations, and after each correction the mirror must be measured to assess the effect of the previous iteration and provide input for the next correction iteration. When using *ex situ* metrology, this means removing the mirror from the IBF plant for measurement, and then replacing it for the next correction. However, to ensure accurate targeting, the mirror must be reproducibly placed back into the IBF system, and any offset in position or rotation must be precisely measured and accounted for.

These two requirements pose conflicting demands on sample handling and mounting. For example, a solution for the IBF mounting is to load the mirror within a mount or holder that can be removed and replaced in the IBF chamber using mechanical stops. However, such an arrangement imposes clamping stresses on the mirror itself that can have an adverse effect on the metrology, particularly measurements of the slope (Siewert *et al.*, 2012 ▸). Even if the mirror is held or clamped under the same conditions, the heating during ion beam figuring and subsequent cooling will cause the sample to expand in the mount, which may have a significant impact on the stress and deformation. This is also one drawback of on-board metrology; the stresses on the mirror in its mounting configuration within the IBF chamber cannot be mitigated, and must therefore be accounted for some other way (in data processing or by careful setup of the measurement), or otherwise considered in the fundamental uncertainties of the metrology.

As discussed, the ideal approach from a metrology perspective is to measure the mirror as close to free-standing as possible. In vertical configurations, where the mirror is lying flat with the active surface pointing upwards, the sag under gravity can be accounted for by modelling the weight and flexibility of the mirror, depending on its dimensions and material composition. However, care must be taken to ensure the mirror is replaced in the exact same configuration in the IBF chamber. This is another area where the use of fiducial marks and an on-board targeting system (such as a camera) can be extremely useful for accurate positioning.

#### 4.2.3. Data processing and frequency filtering

Data processing is not a trivial task, and every transformation applied to data, no matter how small, will impact the results. As discussed, preprocessing steps such as stitching of interferometric measurements must be undertaken with care, and there are often parameters that must be set (such as the tolerance of each stitch, and the minimum overlap between sub-apertures) which affect the final output. When calculating slope data from height data, factors such as the region over which the slope is calculated, and the filter (if any) applied to the data before calculation, can have a significant impact on the final result. As discussed, it is vital to consider these factors when attempting to compare data from two different sources or instruments, where the spatial resolution and instrument transfer function (Groot, 2021 ▸) may differ.

**Figure 11:**
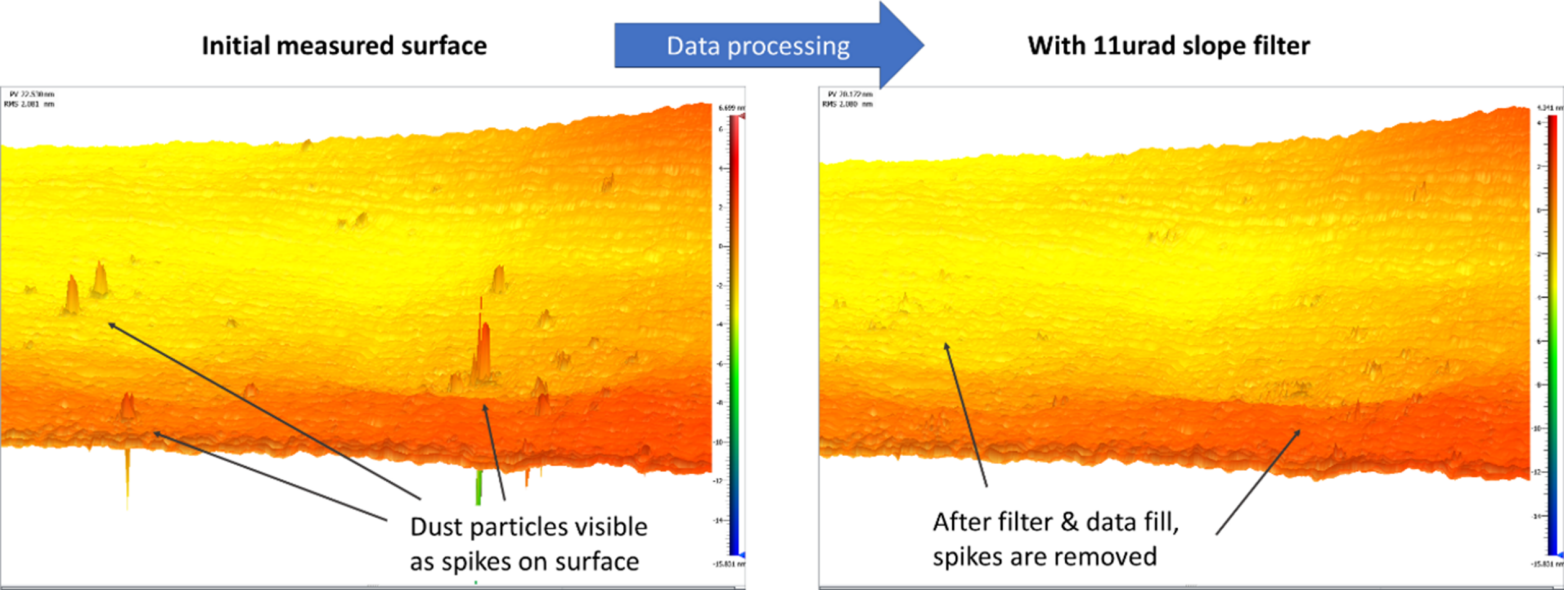
Example of data processing to remove noise, such as spikes caused by particles of dust. Left: measured 3D height profile of a sample, which shows several spikes caused by dust particles or other contaminants. Right: the same surface after applying a slope filter with a threshold of 11 µrad to remove the spikes from the surface.

A consideration for processing of IBF data is the filtering of noise and certain surface features. A common example is the spikes caused in interferometric measurements by dust particles on a mirror’s surface (Fig. 11 ▸). These spikes can be removed by data processing, such as with a spike clip or a slope/height filter. However, this process may risk removal of other, ‘real’ surface features, if the filtering is poorly applied. Another example is the removal of high-frequency features on a mirror surface. As described, the IBF process can only effectively correct errors with the same spatial period as the beam size used. For coarse corrections, it may be more efficient to remove the higher-frequency features that cannot effectively be removed with a given beam size, as this enables more efficient figuring with a lower dwell time and minimal unnecessary removal (Guan *et al.*, 2022 ▸). But this similarly runs the risk of filtering out ‘real’ surface features, if the filtering is not performed in an intelligent or appropriate way.

Ultimately, the result of a measurement is a result of the conditions under which it was taken and the steps that were carried out during processing. It is important that any transformation steps applied are not only well posed but carefully documented and presented alongside the data, to ensure proper comparison and interpretations of the results.

### 4.3. Dwell time calculation and optimization

#### 4.3.1. Choice of algorithms to calculate dwell time

The principle of deterministic figuring is that, given a removal function *B*(*x*, *y*) and a tool dwell time distribution *t*(*x*, *y*), the resulting removal *Z* is obtained through a convolution 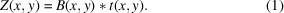
Therefore, when *Z* is the measured height error of a surface that is to be corrected, and *B*(*x*, *y*) is known from characterization of the BRF, the required *t*(*x*, *y*) to correct the height errors can be obtained via inverting the equation and solving the resulting deconvolution between *Z* and *B*.

In practice, solving this deconvolution is non-trivial. Generally, an algorithm is used to optimize the approach and, if necessary, set additional parameters. Most algorithms use either a matrix-based approach, utilizing least-squares minimization to solve the deconvolution (Paige & Saunders, 1982 ▸; Carnal *et al.*, 1992 ▸; Wang *et al.*, 2019 ▸), or an inverse Fourier transform method (Wilson & McNeil, 1987 ▸). Matrix-based algorithms offer improved flexibility, such as the option to consider arbitrary dwell points (Wang *et al.*, 2021*c* ▸), which can be used to minimize tool marks from the IBF process; implementation of dynamic or spatially varying beam effects (Liao *et al.*, 2014*a* ▸,*b* ▸); or simultaneous consideration of multiple figuring tools at once during calculation (such as different sizes of ion beam) (Ke *et al.*, 2022 ▸). However, they are generally computationally expensive, requiring longer calculation times than Fourier transform-based methods. As such, matrix-based methods are generally favoured for 1D figuring (Wang *et al.*, 2019 ▸; Mahji *et al.*, 2024 ▸). In comparison, Fourier transform-based methods, such as the recently developed RIFTA (Wang *et al.*, 2020*b* ▸, 2023*b* ▸), are computationally efficient and able to find effective dwell time solutions in very short calculation times. However, they are generally less flexible than matrix-based methods.

When solving a dwell time deconvolution, the measured data over the clear aperture (CA) (*i.e.* working area of the mirror) should be extended by at least the radius of the tool size to ensure that the CA is worked evenly during figuring. This extended area, known as the dwell grid (DG), may be populated with data generated by various surface extension methods, such as Gaussian, nearest-neighbour or smooth triangular interpolations (Yang *et al.*, 2017 ▸), or by a polynomial fitting to the measured data that is then extended over the DG (Wang *et al.*, 2021*b* ▸). Continuity at the edge of the CA is important to minimize residuals; however, the height of the extrapolated profile outside of the CA should be made low to reduce the total dwell time. Therefore, the optimal surface extension for a given CA typically depends on the nature of the measured surface, the prominence of high- and low-frequency features, and the behaviour of the surface at the boundaries of the CA.

#### 4.3.2. Convergence of both height and slope error

Most figuring methods consider the height error of the mirror under correction. However, slope error is an equally important metric to consider, especially for many X-ray mirrors. Although improving height error will generally also improve slope error, an optimized height profile may yield a sub-optimal slope profile. Given the extremely low slope errors required for X-ray mirrors, it can be challenging to converge to these errors by considering height error alone.

An alternating height/slope optimization method has been shown to efficiently reduce both height and slope errors over 2D surfaces (Wang *et al.*, 2023*a* ▸). This method works by iteratively optimizing first the height error, then the slope error in *x* and *y*, until a solution is found that minimizes both the height and slope errors to the desired accuracy. The 2D slope errors in *x* and *y* of the surface can be obtained from the height profile by simple calculus. Likewise, for a BRF represented by a Gaussian or super-Gaussian function in terms of height removal, the *x* and *y* slope removal profile can be obtained by differentiation. This method was found to be highly effective in correction of slope errors. Using this approach, an elliptical mirror was figured with 0.36 nm r.m.s. height error and 150 nrad slope error over a 150 mm × 20 mm CA (Wang *et al.*, 2023*b* ▸).

## 5. Conclusion

### 5.1. Towards further minimization of height and slope errors

In recent years, IBF has been validated by experimental results as a promising and powerful technique for figure error correction. It can be used on flat or curved surfaces, does not produce edge falloff effects, and is highly compatible with the materials commonly used for X-ray mirrors, such as Si and SiC. The technique is currently used by both commerical manufacturers, and in small in-house facilities, to great success.

The demands of current-generation X-ray mirrors – with sub-nm r.m.s. height errors and sub-100 nrad r.m.s. slope errors over regions hundreds of millimetres long – are within the capabilities of IBF, but are challenging to achieve. The uncertainties associated with every part of the IBF process, including positioning and alignment, motion performance, thermal effects and the stability of removal of the ion beam, must all be carefully assessed and minimized. Likewise, incredibly precise metrology is required to accurately measure such errors. Intelligent data processing, and careful choice of dwell time calculation algorithm, are also essential for enabling correction of figure errors down to the required level.

Development of the IBF process is proceeding particularly fast in the field of academia, where in-house IBF plants at optical labs and synchrotron facilities are pursuing the creation of high-quality, low-figure-error mirrors. As the technology continues to improve and mature, the challenge is to extend the capability of the process to figure errors of <0.1 nm r.m.s. and slope errors of 50 nrad, possibly even 20 nrad. This quality enables diffraction-limited X-ray optical performance. In addition, IBF is uniquely compatible with on-board metrology, which can aid in targeting, allow fast characterization of BRFs from etched craters, and be used to give rapid measurements for coarse figuring. Small in-house IBF plants have been seeing particular success in recent years, and, based on the success of IBF projects at the National Synchrotron Light Source and Advanced Photon Source in the US, the Diamond Light Source in the UK, and the Shanghai Synchrotron Radiation Facility in China, it may be anticipated that more light sources will invest in in-house IBF plants in the coming years.

### 5.2. Recent results from the in-house IBF project at the Diamond Light Source

**Figure 12:**
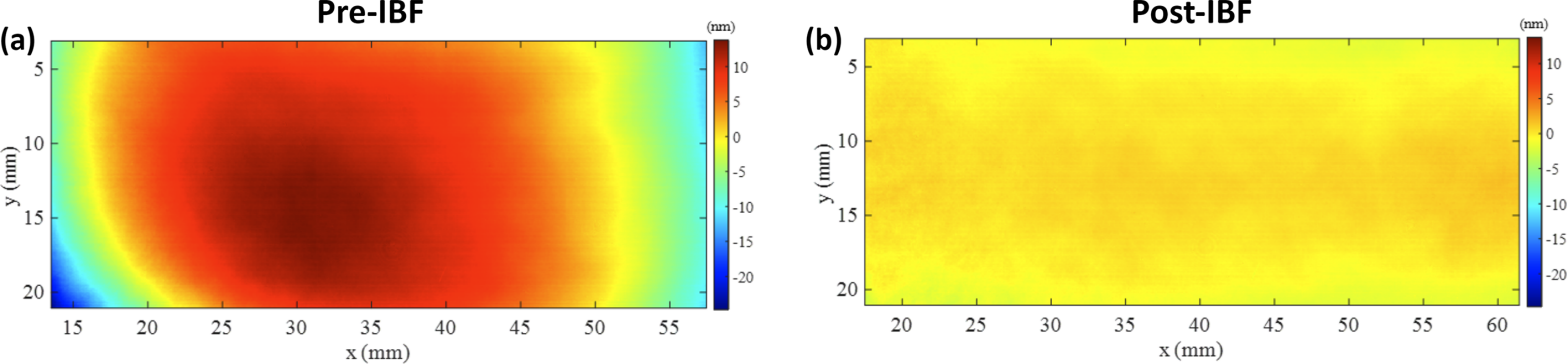
Comparison of the measured height profile of a Si mirror: (*a*) before IBF correction and (*b*) after IBF correction, plotted with the same colour bar. After one iteration of IBF, the PV height error is reduced from 38.5 nm to 5.2 nm, and the r.m.s. height error is reduced from 8.0 nm to 0.76 nm.

The in-house IBF plant in development at the Diamond Light source (Hand *et al.*, 2019 ▸) has demonstrated several promising results in the past year. The figure errors of a trapezoidal mirror with a clear aperture of 95 mm × 20 mm have been efficiently corrected over several iterations (Mahji *et al.*, 2024 ▸). Most recently, sub-nanometre height error and sub-100 nrad slope errors have been achieved over a clear aperture of 44 mm × 18 mm. After one iteration of IBF, the residual height errors were reduced by a factor of >10 (from 8.0 to 0.76 nm r.m.s.), and the tangential slope errors were reduced by a factor of >4 (from 411 to 97 nrad r.m.s.), as shown in Figs. 12 ▸ and 13 ▸.

These results were achieved using a combination of *ex situ* metrology and on-board SAM measurement. *Ex situ* metrology of the samples was performed before and after every iteration of the IBF process to assess the height errors. SAM measurements were used to extract the BRF and removal rate of the ion beam from etched craters. This demonstrated that although the precision of the SAM is limited compared with *ex situ* metrology, it can be used to obtain the BRF parameters, which speeds up the overall figuring process.

Although such results are highly promising, further work remains before the IBF plant can produce mirrors suitable for beamline applications. The next goals of the project are to demonstrate high-quality, sub-100 nrad figuring over a larger clear aperture of 100 mm length; and to demonstrate high-quality figuring of spheric and aspherically curved mirrors (including cylindrical and elliptical). It is hoped that the IBF plant will be ready to deliver beamline-quality mirrors within the next year.

## Figures and Tables

**Figure 13:**
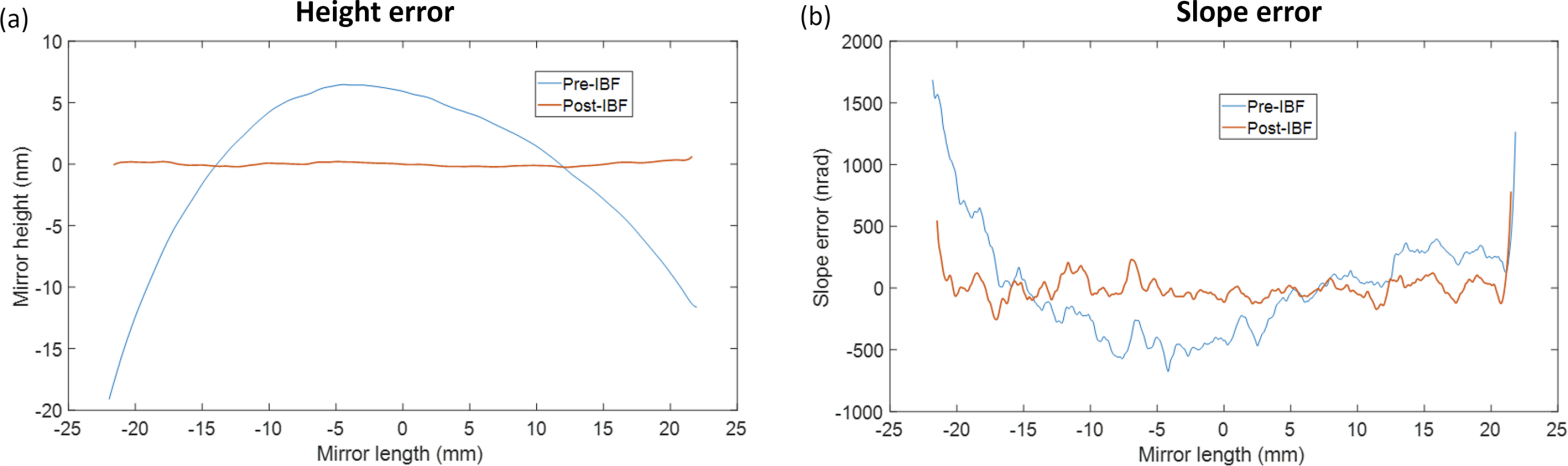
(*a*) 1D line profiles of the sample shown in Fig. 12 ▸ before and after IBF correction. (*b*) Tangential slope error profiles before and after IBF correction, calculated from the height error profiles in (*a*) with a 2 mm filter applied. The r.m.s. slope error is reduced from 411 nrad to 97 nrad after one iteration of IBF.

## Appendix A List of sources for Fig. 4

2D figuring data sourced from: Wilson & McNeil (1987 ▸); Allen & Romig (1990 ▸); Allen *et al.* (1992 ▸); Allen (1995 ▸); Drueding *et al.* (1995 ▸); Schindler *et al.* (2003 ▸); Hänsel *et al.* (2007 ▸); Franz & Hänsel (2010 ▸); Demmler *et al.* (2010 ▸); Wang *et al.* (2020*a* ▸); Wang *et al.* (2021*b* ▸); Wang *et al.* (2021*c* ▸); Wang *et al.* (2023*a* ▸); Wang *et al.* (2023*b* ▸).

1D figuring data sourced from: Idir *et al.* (2015 ▸); Zhou *et al.* (2016*b* ▸); Wang *et al.* (2019 ▸).
